# Esmolol reduces anesthetic requirements thereby facilitating early extubation; a prospective controlled study in patients undergoing intracranial surgery

**DOI:** 10.1186/s12871-015-0154-1

**Published:** 2015-11-28

**Authors:** Irene Asouhidou, Anastasia Trikoupi

**Affiliations:** Department of Anesthesiology “G.Papanikolaou” General Hospital, 15-17 Agiou Evgeniou Street, 55133 Thessaloniki, Greece

**Keywords:** Craniotomy, Esmolol, Neuroanesthesia, Propofol, Sevoflurane

## Abstract

**Background:**

Adequate cerebral perfusion pressure with quick and smooth emergence from anesthesia is a major concern of the neuroanesthesiologist. Anesthesia techniques that minimize anesthetic requirements and their effects may be beneficial. Esmolol, a short acting hyperselective β-adrenergic blocker is effective in blunting adrenergic response to several perioperative stimuli and so it might interfere in the effect of the anesthetic drugs on the brain. This study was designed to investigate the effect of esmolol on the consumption of propofol and sevoflurane in patients undergoing craniotomy.

**Method:**

Forty-two patients that underwent craniotomy for aneurysm clipping or tumour dissection were randomly divided in two groups (four subgroups). Anesthesia was induced with propofol, fentanyl and a single dose of cis-atracurium, followed by continuous infusion of remifentanil and either propofol or sevoflurane. Patients in the esmolol group received 500 mcg/kg of esmolol bolus 10 min before induction of anesthesia, followed by additional 200 mcg/kg/min of esmolol. Monitoring of the depth of anesthesia was also performed using the Bispectral Index-BIS and cardiac output. The inspired concentration of sevoflurane and the infusion rate of propofol were adjusted in order to maintain a BIS value between 40–50. Intraoperative emergence was detected by the elevation of BIS value, HR or MAP.

**Results:**

The initial and the intraoperative doses of propofol and sevoflurane were 18–50 mcg/kg/min and 0.2–0.5 MAC respectively in the esmolol group, whereas in the control group they where 100–150 mcg/kg/ and 0.9–2.0 MAC respectively (*p* = 0.000 for both groups). All procedures were anesthesiologically uneventful with no episodes of intraoperative emerge.

**Conclusions:**

Esmolol is effective not only in attenuating intraoperative hemodynamic changes related to sympathetic overdrive but also in minimizing significant propofol and sevoflurane requirements without compromising the hemodynamic status.

ClinicalTrials.gov Identifier: NCT02455440. Registered 26 May 2015.

## Introduction

Neurosurgical procedures result in a significant release of adrenaline requiring large amounts of anesthetics and analgesics. However all agents used in neurosurgery should have minimal effect on brain function, not interfere with intraoperative neuromonitoring, and allow for a quick and smooth extubation and recovery. Rapid emergence from anesthesia after tumor dissection or aneurysm clipping is essential for early detection of possible neurological impairment due to hematoma formation, edema or vasospasm and ischemia. In addition, early recovery and extubation is associated with fewer metabolic and cardiovascular changes than a 2-hour delayed recovery [1].

Anesthesia with either propofol or sevoflurane combined with a short acting opioid, like remifentanil, is the preferred pharmacological approach in neuroanesthesia. Propofol and sevoflurane have a quite similar recovery time and neuroprotective effect but still controversy exists regarding the best choice of anesthetics for the neurosurgical patient [2, 3]. Although propofol is an appropriate component of total intravenous neuroanesthesia, in prolonged neurosurgical cases there is always the risk of delayed awakening. On the other hand, the use of sevoflurane in neurosurgery is still under consideration due to its ability to provoke vasodilation, cerebral hyperemia and increase intracranial pressure (ICP) even in the subanesthetic dose of 0.4 MAC (minimum alveolar concentration) [1, 4–6]. Investigators have reported significantly high doses of propofol (75–200 mcg/kg/min) and sevoflurane (2.5 MAC) intraoperatively during craniotomy maintaining a BIS (Bispectral Index) value of 40–50, which might delay the recovery time [3, 7–11]. These facts limit the use of both these anesthetic agents.

Esmolol is an ultra-short acting, cardioselective β_1_-adrenergic receptor antagonist that is effective in blunting sympathetic overdrive to several perioperative stimuli, including laryngoscopy with intubation, intraoperative events, emergence, and extubation and can be used as an alternative to opioids for maintaining hemodynamic and BIS stability during general anesthesia [12]. Esmolol enhances the analgesic effect of opioids but has no analgesic effect when administered alone [13, 14]. As a β_1_-adrenergic blocking agent, esmolol is not a cerebral vasodilator and has no significant effect on ICP [15, 16]. Studies evaluating the interaction between β-adrenoceptor antagonists and anesthetics on BIS have concluded that esmolol minimizes the dose of propofol required for intubation [12, 17]. Our study investigates the effect of esmolol on intraoperative requirements of propofol and sevoflurane in patients undergoing craniotomy.

## Methods

The local research ethics committee (Scientific and ethics committee of G.Papanikolaou Hospital) approved the study and written informed consent was obtained from all patients. We included in the study ASA I-II patients, aged from 18 until 75 years, scheduled for resection of brain tumor or aneurysm clipping under general anesthesia. Exclusion criteria included the presence of one or more of the following: ASA > II, Body Mass Index (BMI) > 30, indication for rapid sequence induction, contraindication to β-blocker administration, atrial fibrillation, Glasgow Coma Scale (GCS) < 15, history of drug abuse, preoperative aphasia, neurologic deficit or preoperatively foreseen delayed extubation.

The patients were randomly divided in two groups; the esmolol group and the control group. Patients in the control group were further divided in two subgroups: control-propofol and control-sevoflurane. Similarly, patients in the esmolol group were further divided in two subgroups: esmolol-propofol and esmolol-sevoflurane. The method used for randomization was simple randomization, similar to repeated fair coin-tossing. This method was used also for randomly allocating TIVA vs sevoflurane too.

Before induction in anesthesia standard monitoring (electrocardiogram of five leads-ECG, noninvasive arterial blood pressure and pulse oximeter probe) was placed. Peripheral vein access was established and an isotonic crystalloid solution 10 ml/kg before induction and 5 ml/kg/h intraoperatively was infused. All patients had a 20G catheter inserted in the radial artery for invasive monitoring of blood pressure and cardiac output (CO) using arterial pulse contour analysis. Cardiac output was calculated using the FloTrac™ sensor kit connected to the arterial line and to the Vigileo™ monitor programmed with the 3.02 version (Edwards Lifesciences 2009, Thessaloniki, GR) of the software for this device. Patient data (age, gender, body weight, and height) were entered and after checking the arterial line waveform fidelity, the system was zeroed and CO measurement was initiated.

A BIS sensor (Aspect Medical System, Inc, GR) was placed in the frontal area with consideration for the location of the surgical incision. Regional brain hemoglobin oxygen saturation (rSO_2_) was also monitored using near-infrared spectroscopy. Immediately before induction of anesthesia, radiolucent Adult Somasensor oximetry strips were applied bilaterally to the forehead at standard positions relative to the midline. Consideration was also given to the site of craniotomy. The somasensors were connected to an INVOS™ cerebral oximeter, Model 4100 (Somanetics Corp, Covidient, Greece), which provides real-time numerical rSO_2_ values for bilateral frontal cortex. An initial baseline measurement of rSO_2_ was performed bilaterally by taking the mean of readings of the last 10 min before intubation. Intraoperative normothermia was actively maintained with a forced airwarming blanket.

Esmolol group patients received 500 mcg/kg of esmolol 10 min before the induction in anesthesia and for a period of 4 min followed by continuous infusion of 200 mcg/kg/min. General anesthesia was induced with propofol (2 mg/kg) and fentanyl (2mcg/kg). Intubation of the trachea was facilitated by cis-atracurium (0.15 mg/kg) and ventilation was adjusted to an end-tidal-carbon dioxide (EtCO_2_) tension of 30–35 mmHg in 50 % O_2_/air. Anesthesia was maintained using remifentanil (0.2 mcg/kg/min) and either propofol or sevoflurane. The inspired concentration of sevoflurane and the infusion rate of propofol were adjusted in order to maintain BIS values between 40–50. Intraoperative emergence was detected by elevation of BIS value, HR or MAP. After extubation, patients were asked if they had experience episodes of recall. The use of neuromuscular block was avoided after intubation. At dura closure all patients received paracetamol 1gr. Immediately before the discontinuation of remifentanil, all patients received 1 mcg/kg of fentanyl. Propofol or sevoflurane and remifentanil were discontinued after skin closure while the administration of esmolol was continued for an additional 30 min following extubation. The dose of esmolol in the esmolol group was reduced in cases of bradycardia (heart rate-HR < 40) and in cases where mean arterial pressure-MAP < 50 mmHg. Patients in the control group, who did not receive esmolol, were transferred to the intensive care unit (ICU) where they were extubated in the following hours. This decision was made because it was considered safer for the patients not to be extubated immediately after the end of the procedure in order to avoid any increase of blood pressure, considering that they had not received β-blocker for sympathetic stimulus control.

## Study endpoints

Parameters recorded were operative time, duration of anesthesia (time from induction till discontinuing of remifentanil-propofol/sevoflurane), orientation time and extubation time. Orientation time was defined as the time from discontinuation of all anesthetic agents until the time the patient opened his eyes and performed specific movements. Extubation time was defined as the time from drug discontinuation to extubation. The time to reach an Aldrete score of at least 9 after tracheal extubation was also recorded. The Aldrete scoring system is widely accepted in order to assess the physical status of patients recovering from anesthesia based on a score of five criteria (mobility-able to move 4 extremities on command, respiration-able to breathe deeply and cough, oxygenation-O_2_ saturation on room air > 92 %, cardiovascular stability-MAP stable and consciousness-fully awake) [18].

## Statistics

Data were expressed as mean ± SD. Differences in categorical data were evaluated using the student t test. A beta error level of 20 % or statistical power of 80 % and a α-level of 0.05 was used to calculate the sample size of this study. A value of *p* < 0.05 was considered to represent statistical significance.

## Results

A total of 42 patients (male/female: 23/19, mean age 53.3 ± 17.24) were enrolled. There were no significant differences between the two groups in regard to their demographics, duration of anesthesia or type of surgery (Table 1). All the patients enrolled completed the study.

**Table 1:** Demographics of patients and perioperative data

	Control group	Esmolol group	*P* value	Total
Number of patients	21	21		42
Body weight (kg)	76.00 ± 10.86	82.4 ± 16.62	>0.05	77.7 ± 14.13
Sex (M/F)	12/9	11/10	>0.05	23/19
ASA (I/II)	11/10	9/12	>0.05	20/22
Operation				
Tumor dissection	15	15		30
Aneurysm clipping	6	6		12
Operative time (min)	267.4 ± 87.52	234.4 ± 74.06	>0.05	241.4 ± 82.034
Anesthesia duration (min)	345.2 ± 51.08	303.8 ± 84.17	>0.05	338.0 ± 91.54
Orientation time (min)	-	8.4 ± 3.88		
Extubation time (min)	-	15.6 ± 6.98		

**Fig. 1:**
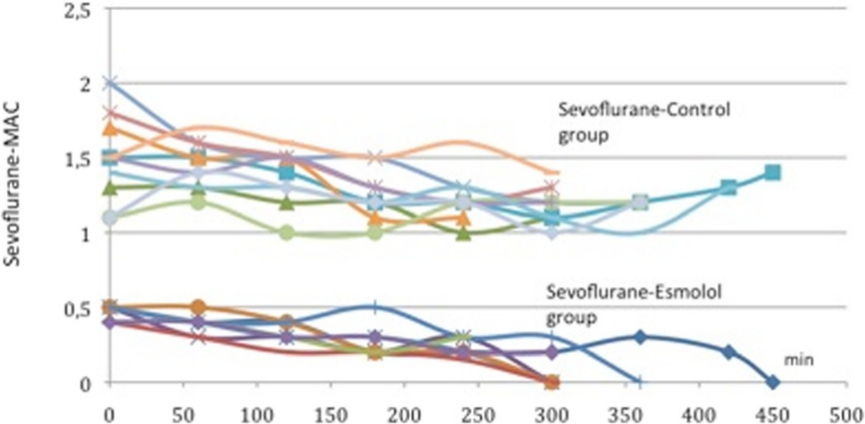
Intraoperative fluctuations of propofol in the control (top of the picture) and the esmolol group (bottom of the picture). Each patient is indicated with a different color. Initial infusion rate of propofol is higher in the control group compared to the esmolol group. Also, infusion rate of propofol at each time point was much lower for the esmolol group than for the control group. Mean value of propofol in control group was 134 ± 12.9mcg/Kg/min, where in the esmolol group was 25.83 ± 9.32 mcg/kg/min during the procedure

**Fig. 2:**
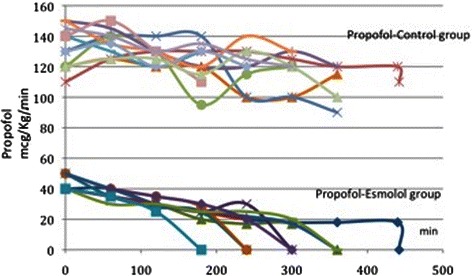
Intraoperative fluctuations of sevoflurane in the control (top of the picture) and the esmolol group (bottom of the picture). Each patient is indicated with a different color. Initial MAC of sevoflurane is higher in the control group compared to the esmolol group. Also MAC of sevoflurane at each time point was much lower for the esmolol group than the control group. Mean value of sevoflurane in control group was 1.49 ± 0.288 MAC MAC, where in the esmolol group was 0.31 ± 0.13MAC during the procedure

In the control-propofol group (11 patients), propofol infusion started at rates ranging from 100 to150 μcg/kg/min (134 ± 12.9mcg/Kg/min) and the infusion rate for each patient remained quite stable throughout the procedure. Conversely, in the esmolol-propofol group (11 patients), propofol was initiated at 40mcg/kg/min in all patients and was reduced to 18-35 μcg/kg/min (25.83 ± 9.32 mcg/kg/min) during the procedure (*p* = 0.000) [Fig. 1]. In the control-sevoflurane group (10 patients), sevoflurane’s MAC was initiated at 0.9 to 2 MAC (1.49 ± 0.288) and remained quite stable throughout the procedure with no significant fluctuation in any patient. Conversely, in the esmolol-sevoflurane group (10 patients) the initial MAC of sevoflurane was 0.5 MAC for all patients and was adjusted at 0.2–0.3 MAC (0.31 ± 0.13) during the operation (*p* = 0.000) [Fig. 2]. It should be noted that BIS values were comparable between the control and the esmolol group considering that BIS values cannot be absolutely stable. We adjusted propofol infusion rate and inspired concentration of sevoflurane in order to maintain a BIS between 40–50 throughout the procedure.

**Fig. 3:**
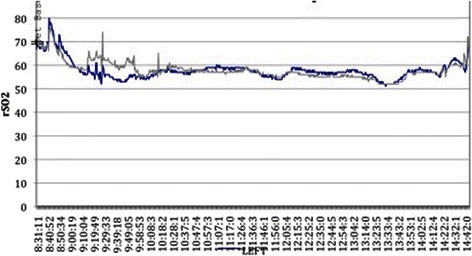
Desaturation of regional cerebral oxygenation during craniotomy. The blue arrow indicates the period of desaturation

All patients in the esmolol group were successfully extubated with excellent neurological outcomes, coinciding with the data obtained from the intraoperative clinical data of the patient, the regional brain saturation monitoring and the macroscopic estimation of the condition of the cerebral tissue. In one patient in the esmolol group there was extensive bleeding during tumor dissection resulting in an hemoglobin drop from 12.3 to 9.8 mg/dl without however any significant change in cardiac output. However, the value of the right rSO_2_ revealed desaturation 26 %, even though the hemoglobin at that moment was not very low (Fig. 3). The patient was transfused with two packs of red blood cell (600ml) and the value of rSO_2_ returned to normal. The patient was successfully extubated 7 min after the end of the operation.

In the esmolol group, orientation and extubation times were 8.81 ± 4.69 min and 14.4 ± 6.84 min respectively for the propofol subgroup and 7.57 ± 2.82 min and 14.1 ± 6.99 min respectively for the sevoflurane subgroup. There were no significant differences between the two subgroups in orientation times (*p* = 0.553) and extubation times (*p* = 0.949). However, there was a trend towards sevoflurane achieving a better and more rapid emerge, although orientation times did not significantly differ between the propofol and sevoflurane group. The Aldrete score criteria were reached before extubation, so the actual time of the Aldrete score of at least 9 was 0 min for all patients in the esmolol group.

All patients in the esmolol group were fully conscious with no hypoxia after extubation. No patient experienced hypertension at the time of extubation or during the early postoperative period after the discontinuation of esmolol. Hypertension defined as an increase in SBP > 20 % of the baseline.

There was only one case of postoperative shivering. There was not noticed any episode of intraoperative emerge, that could be attributed to low dose of sevoflurane and propofol. This was in agreement also with the values of BIS. Moreover, none of the patients reported any episode of recall after when they were asked at the first postoperative day.

**Fig. 4:**
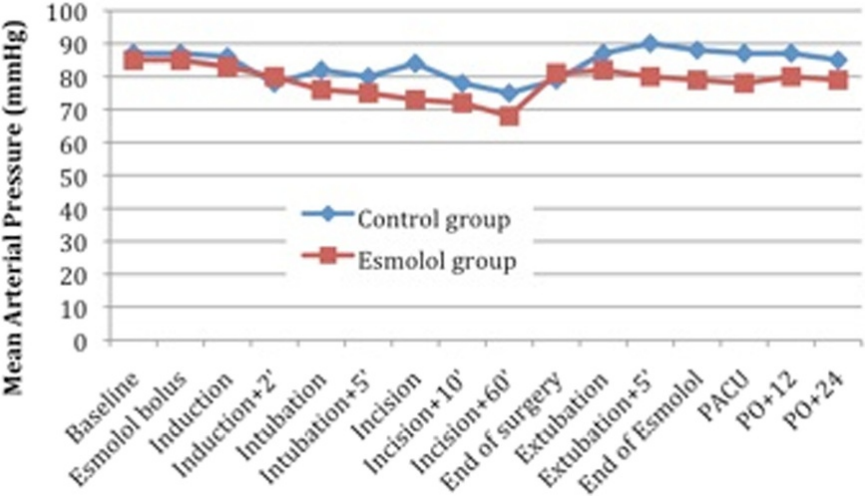
Mean arterial pressure (MAP) fluctuation regarding perioperative time in the control and esmolol group

**Fig. 5:**
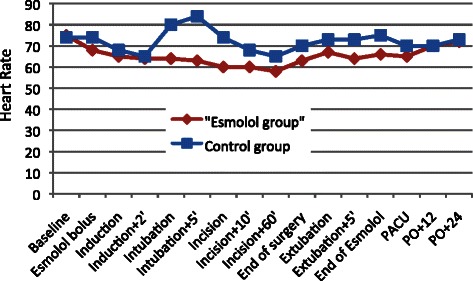
Heart rate fluctuations during perioperative period in the control and esmolol group

The esmolol regimen used in the present study did not have any severe hemodynamic effect during induction or maintenance of anesthesia. Predosing with esmolol 10 min before induction in anesthesia did not affect the BIS index value. There was no episode of bradycardia or persistent hypotension requiring intervention. Cardiac output did not decrease significantly even though MAP and HR were decreased. The esmolol group showed lower overall HR than the control group. Heart rate during tracheal intubation, skin incision, and tracheal extubation fluctuated less in the esmolol group. The MAP and HR variations during anesthesia are depicted in Figs. 4 and 5, respectively. There was no significant difference in HR and MAP between the control and esmolol group before induction in anesthesia. However after administration of esmolol, MAP and HR was significant different; from beginning of infusion until the first postoperative period, mean MAP at the control group was 83.8 ± 4.46mmHg when for the same period at the esmolol group was 78.5 ± 4.7mmHg (*p* = 0.003). Also, for the same period, mean HR at the control group was 72.2 ± 4.99/min when at the esmolol group was 65.2 ± 4.45/min (*p* = 0.000).

## Discussion

One of the major challenges of neuroanesthesia is the ability to provide effective intraoperative analgesia and anesthesia, perioperative hemodynamic stability and smooth and early extubation. Our study showed that esmolol significantly decreased the consumption of anesthetics (propofol and sevoflurane) for patients undergoing elective craniotomy [7–11]. This reduction in anesthetics is clinically important because it achieves early orientation, less recovery time, and a smooth extubation without any hypertension during extubation. Moreover the administration of esmolol allows for the use of any anesthetic agent in “subanesthetic” doses (<0.5 MAC). Administration of esmolol before induction in anesthesia with propofol did not affect BIS suggesting that esmolol does not modify BIS during general anesthesia. On the one hand many authors argue that this is the result of the lack of any substantial sympathetic activation before intubation [19, 20]. On the other hand patients suffering from a cerebral tumor or even an unruptured aneurysm, have their sympathetic system already stimulated [21].

In the current study, the mechanism by which esmolol decreased anesthetic requirements is unknown. Miller et al. [22] suggested that drugs that affect central catecholamine release might alter anesthetic requirements. Our results clearly indicate that β-adrenoceptor antagonists not only block cardiovascular stress responses after noxious stimulation, but also increase the antinociceptive component of anesthesia. Esmolol, unlike propranolol [23], has no analgesic activity, is believed to have minimal sedative effects and given its low lipid solubility does not cross the blood–brain barrier in significant amounts [20, 24].

One explanation for the action of esmolol on central antinociceptive mechanism is its propensity to block β-adrenoceptors. The hemodynamic effects of esmolol are thought to be mediated by the blockage of peripheral, β-adrenergic receptors [25]. B-adrenergic receptors are present in various parts of the reticular activating system, particularly the medial septal region of the basal forebrain. Administration of β-adrenergic receptor agonists in this region elicits enhancement of behavioral and EEG indices of waking in animals [26]. The low-potency, low-lipid solubility and rapid metabolism within the blood stream do not exclude a central site for esmolol action [27].

Many previous studies have focused on the relationship between perioperative use of β-blocker with opioids. However, very few have addressed the interaction between β-blockers and anesthetics agents. Wilson et al, using esmolol in patients before intubation found that the propofol requirements for induction of anesthesia were reduced by 25 %. Based on those findings he suggested that there might be a correlation between reduced requirements of propofol and low CO attributed to the use of esmolol. However, in this study there was no CO monitoring during esmolol infusion [28]. In another study on patients undergoing non-neurosurgical procedures, it was shown that high doses of esmolol (250mcg/kg/min) reduced propofol requirements by 26 %. The authors proposed that, since there was no evidence of altered propofol pharmacokinetics, esmolol interacted with the opioid component in an unspecified manner [29].

Esmolol is thought to suppress the EEG, with a decrease in BIS and an increase in the burst suppression ratio during propofol/alfentanil anesthesia [23]. However this is in contrast with a recent study reporting that the blood concentration of propofol necessary to preventing response to command was unaltered by esmolol. According to Orme et al esmolol may alter drug metabolism and distribution via effects on hepatic blood flow and subsequent drug clearance [20]. In addition, redistribution of propofol from the central compartment may be limited and the decreased consumption of anesthetics may be due to decreased CO and consequently decreased hepatic blood flow altering the pharmacokinetic of propofol [24]. However, this is not in agreement with our findings that CO was not changed during infusion of esmolol. Concurrent with our results is also the one by Chia et al. [30] reporting that MAP was not different between groups that received esmolol and those that did not receive. Thus it seems unlikely that the reduction in CO is responsible for the low MAC of sevoflurane and propofol. Moreover, esmolol is metabolized by red blood cells esterases and not by the liver.

Adequate cerebral blood flow needs adequate global hemodynamics including blood pressure and CO. The major concern about esmolol is the possible bradycardia and low MAP that may occur following its administration. Regional cerebral oxygen saturation and CO monitoring by INVOS™ and FloTrac™ closely monitor cerebral perfusion and oxygenation respectively in patients who receive esmolol. Our results indicate that esmolol infusion did not produce significant changes in HR or CO, indicating that esmolol even in the dose of 200mcg/kg/min, is a safe component of balanced anesthesia during craniotomies.

Another issue that may arise is that the low dose of anesthetics agents used intraoperative might lead to a higher cerebral metabolic ratio of oxygen (CMRO_2_). However the values of rSO_2_ did not show any reduction that could correlate with inadequate level of anesthesia.

Certain limitations of this study have to be recognized although we consider that they did not alter the accuracy of our results. The study population was relatively small, and we did not use target controlled infusion (TCI) for calculating the dose of propofol and remifentanil.

## Conclusion

Our data revealed that continuous esmolol infusion could significantly decrease anesthetic requirements during balanced anesthesia with propofol or sevoflurane and remifentanil, resulting in successful and uneventful emergence, with no significant changes in heart rate or cardiac output. B-adrenergic antagonists may represent a novel class of drugs that can modify anesthetic requirements in patients undergoing craniotomy and be component of balanced anesthesia.
